# Perceived opportunities of clinical reasoning learning in postgraduate psychiatry training: Trainees’ and faculty’s perspectives

**DOI:** 10.5339/qmj.2024.14

**Published:** 2024-04-03

**Authors:** Dalia Albahari

**Affiliations:** 1 Hamad Medical Corporation, Doha, Qatar Email: dalbahari@hamad.qa

**Keywords:** clinical reasoning, psychiatry residency, psychiatry clinical fellowship, faculty

## Abstract

Background: Learning clinical reasoning is less effective in isolation of clinical environments because contextual factors are a significant component in the clinical reasoning process. This study investigated the differences in opinions between novice and expert clinicians on learning clinical reasoning in the workplace.

Materials and Methods: The author used a cross-sectional online survey design to investigate the perceived learning of six clinical reasoning skills in 13 learning opportunities. Questionnaires were emailed to 41 postgraduate psychiatry trainee doctors and 37 faculty members. Data were analyzed descriptively. The Chi-square test was used to compare the responses of the two groups. Statistical significance was set at *P* < 0.05.

Results: The combined response rate was 73.07%. The two groups perceived the learning of advanced clinical reasoning skills to be lower than that of basic skills. There were significant differences in the perceived learning of basic clinical reasoning skills in self-study/exam preparations (*P* = 0.032), general hospital grand rounds (*P* = 0.049), and clinical rounds (*P* = 0.024 for consultant-led rounds and *P* = 0.038 for senior peer-led rounds). There were also significant differences in the perceived learning of advanced clinical reasoning skills among peer-led tutorials (*P* = 0.04), journal clubs (*P* = 0.006), morning reports (*P* = 0.002), and on-call duties (*P* = 0.031).

Conclusions: The trainees showed a significant preference for structured learning environments rather than clinical environments, especially for advanced clinical reasoning skills. Trainees likely struggled with cognitive overload in clinical environments. Local postgraduate psychiatry programs will likely benefit from implementing multiple educational interventions that facilitate teaching and learning clinical reasoning in complex clinical environments.

## Background

Expert clinicians demonstrate superior clinical reasoning (CR)^1^ skills after extensive clinical exposure.^1,2^ Advanced knowledge organization in long-term memory facilitates non-analytical reasoning by experts through pattern recognition.^3–5^ While novices mostly use hypothetic-deductive reasoning, a lengthy and more exhaustive method operates through working memory.^5–7^ Working memory is essential for information processing and organization; however, its limited capacity can be easily exhausted, leading to cognitive errors.^8–10^ Contextual factors influence the cognitive CR processes of experts and novices.^11^ The contextual factors lead to increase in cognitive load complexity, and this may dictate a shift between analytical and non-analytical clinical reasoning processes.^12,13^ Contextual factors were found to affect the CR of all clinicians, most likely through cognitive overload. However, less experienced doctors were more vulnerable to cognitive errors, diagnostic uncertainty, and negative emotional reactions.

As a phenomenon, CR complexity further complicates learning. CR includes multiple sub-skills that stretch between diagnosis and management.^14,15^ This study examined six CR skills based on the CR curriculum developed by Harendza et al.^16^

Information gathering (IG)^2^ is the first CR skill that trainees learn. It provides a CR start-up and directs the process.^17^ IG skills have two components: clinical information content, which forms knowledge schemas, and the process of gathering such information, which influences schema organization, information retrieval, and pattern recognition.^17,18^ Although content knowledge is often the focus of formal teaching and learning, the process is also important. An IG process that is responsive to clinical cues provides a suitable medium for managing complex clinical knowledge and generating accurate hypotheses. Thus, IG, the management of the information gathered, and the testing of clinical hypotheses run simultaneously and should be taught as dynamic rather than linear processes.^19^

Pattern recognition skills (PR)^3^ develop through experience. However, early training in PR is desirable because using both types of dual processing is associated with a more accurate diagnosis among novices.^20^

Cost-effective resource management (CE)^4^ is another advanced CR skill. Multiple initiatives aim to integrate leadership and management training into clinical training to meet the expectations of clinicians demonstrating management skills at various levels.^21–23^

Evidence-based practice (EB)^5^ is another advanced CR skill that, if mastered, empowers doctors to deliver safer and more patient-centered care.^24–26^

Learning clinical reasoning in the postgraduate clinical environment can be challenging as healthcare services prioritize patient care. Modern medical curricula incorporate several educational strategies to teach CR on the job, such as deliberate practice, diverse contextual exposure, coaching, and stress management training.^27–30^ Without a designated CR curriculum, clinical teachers may face the challenge of implementing these educational strategies in health service activities with limited flexibility and significant contextual factors. No specific intervention has proved superior in specific clinical environments. Deliberate practice remains the most valid method for effectively learning CR, but faculty and learners must understand the complexity of deliberate practice.^5^

### Postgraduate psychiatry training in Qatar

The disabilities caused by mental health disorders are a global concern.^31,32^ Qatar is no exception, with the prevalence of mental health disorders among the Qatari population reaching 20%.^33^ Due to the critical role of well-developed mental health services, Qatar prioritized mental healthcare as a national health strategy.^34^ Education and training in psychiatry are one of the pillars for service enhancement and excellence in patient care. Mental Health Services of Hamad Medical Corporation is the sole provider of public mental health services and the only institute that provides postgraduate psychiatry training programs in Qatar. The postgraduate psychiatry residency program has developed significantly since it was established in 1994. Since then, the program has been continuously improving, forming collaborations with the Arab Board of Health Specializations, Weil Cornel Medicine-Qatar, and the College of Medicine at Qatar University. It also received accreditation from the Accreditation Council of Graduate Medical Education- International division (ACGME-I)^6^ in 2013. The psychiatry postgraduate residency program is a four-year program. Trainees may pursue a higher level of subspecialized training (clinical fellowship) after graduating from the residency program. The clinical fellowship is a three-year program that prepares trainee doctors for their future roles as consultant psychiatrists. The psychiatry trainees are supervised by consultant psychiatrists (faculty members). The residency program follows ACGME^7^ competency framework, and the clinical fellowship program follows a locally developed curriculum. None of the curricula included CR as a competency when the research was conducted in January 2020. The lack of CR curricula may comprise an educational gap that weakens training in this area. Multiple contextual factors like the medical hierarchy system, emotions, and certain environmental factors were found to act as a hidden curriculum for learning CR in Qatar’s postgraduate psychiatry programs.^35^ Hidden curricula may negatively influence CR learning.^36^

This study aimed to compare the opinions of experts (consultant psychiatrists) and novices (postgraduate psychiatry residents and clinical fellows) on their perceived learning of various CR skills through educational and service activities in local postgraduate psychiatry training programs, considering the lack of CR curricula. The results provide insights into perceived opportunities for learning CR from two perspectives in the current structure of local mental health services.

## Materials and Methods

A cross-sectional survey was conducted in the Mental Health Services Department of Hamad Medical Corporation in Qatar. Online questionnaires were administered to postgraduate psychiatry trainee doctors and faculty members.

### Study Design and Setting

This cross-sectional online survey was conducted among postgraduate psychiatry residents, clinical fellows, and faculty members of the Mental Health Services Department of Hamad Medical Corporation in Qatar. Mental Health Services is the sole provider of public mental health services and the only institute that provides postgraduate psychiatry training programs in Qatar.

### Sample

The population studied included postgraduate psychiatry residents, clinical fellows, and faculty members (consultant psychiatrists) of the Mental Health Services Department of Hamad Medical Corporation in Qatar. The total population sample was used because the number of targeted participants was relatively small (41 residents and fellows and 37 faculty members). The only demographic data collected were job titles to ensure that the survey covered a spectrum of clinical experiences. Previous formal faculty training or experience in CR teaching was not investigated.

### Theoretical Framework

Cognitive psychology and social cognitive theories, including situated cognitive and distributed cognitive theories, were used as the framework for this study. Cognitive psychology theories improve the understanding of how information is processed, stored in schemas, and retrieved based on clinical cues.^4,5,37^ These theories also help understand the cognitive development of novices from basic knowledge to illness script to experiential knowledge.^38^ Cognitive psychology theories have been criticized for viewing CR as a mental process isolated from its context. Conversely, social cognitive theories emphasize the dynamic bidirectional relationship between learners and their social and physical environments.^39,40^ Social cognitive theories connect learning to the context in which it occurs and value collective cognition in a particular environment.^40–42^

This study used cognitive psychology and social cognitive theories to examine the research question using a positivist paradigm. This framework allowed us to understand the impact of various contexts on CR cognition and learning from two perspectives.

### Research Tool

The author developed two anonymous 5-point Likert scale questionnaires (Appendices 1 and 2). The questions were designed based on the author’s knowledge of the local healthcare system, the CR training model of Harendza et al.,^16^ and broader evidence from the literature. Questionnaire 1 targeted psychiatry residents and Questionnaire 2 targeted faculty. The questionnaires assessed the respondents’ perceptions of the value of the 13 learning opportunities (LOs)^8^ in facilitating the six CR skills. The questions investigated the same LOs and CR skills but were phrased slightly differently to suit the target group. The questionnaire responses ranged from *very likely* to *very unlikely*. A *not-applicable* answer was added to Questionnaire 1 as junior residents do not work in outpatient clinics.

#### Variables

##### i. Level of experience

The only demographic data collected were job titles, which reflected clinical experience levels.

##### ii. Clinical reasoning skills

Based on the Harendza et al.^16^ curriculum, the author divided the CR skills into basic and advanced skills (see Table 1).

##### iii. Learning opportunities

The author identified 13 learning opportunities that were part of either a formal residency educational program or mental health service delivery.

The identified learning opportunities fell under three groups.

(1) Classroom learning opportunities: morning reports, case presentations, peer-led tutorials, journal clubs, and general hospital grand rounds.
(2) Clinical learning opportunities: consultant-led ward rounds, senior peer-led ward rounds, multidisciplinary team meetings, on-call duties, and outpatient clinics.
(3) Informal learning opportunities: informal discussions with consultants, informal discussions with peers, and self-study/exam preparations.

For a detailed description of the learning opportunities, please refer to Table 2.

All trainee doctors had access to all learning opportunities except for the first- and second-year residents, who did not run outpatient clinics.

The questionnaire’s validity was tested by face and content validity. Details of the questionnaires’ validation process are available from the author upon reasonable request.

### Data Collection Procedure

The author used the SurveyMonkey website^43^ to distribute the questionnaires using the participants’ work emails obtained from the administrative staff of the training programs. In January 2020, the author sent personalized invitation emails containing the participant information sheet and a link to the online questionnaires. Reminder emails were sent twice. The first reminder email was sent after two weeks, and the second was sent four weeks after the original invitation emails. Consent to participate was considered implicit when the questionnaire was completed.

### Data Analysis

The data were entered into the STATA 15 software, analyzed descriptively, and reported as percentages. As there were missing responses to some of the questions, the responses were calculated against each question’s total responses and not the entire sample. The Chi-square test was used to compare the trainees’ and faculty members’ five-scale responses (categorical data), and a significance level of *P* < 0.05 was set. The *very likely* and *somewhat likely* responses were combined and reported as favorable ratings. None of the other categories were reported.

## Results

The combined response rate was 73.07%. Thirty-six (87.80%) out of 41 trainee doctors and 21 (56.75%) out of 37 consultant psychiatrists completed the questionnaires. The respondents represented a satisfactory spectrum of clinical experience according to their job titles. There were 36.85% consultant psychiatrists, 33.32% clinical fellows, and 29.83% residents.

### Basic CR Skills Learning in the Identified Learning Opportunities

Trainees and consultants had relatively lower ratings for GH than IG and CCK.

**Figure 1.**
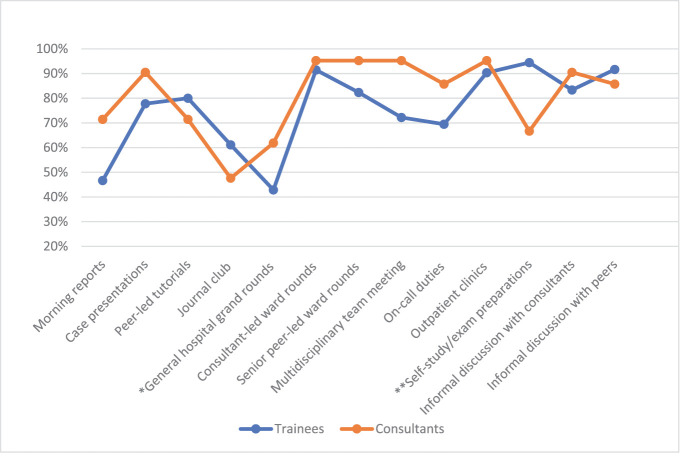
Information gathering skill - favorable ratings. *The consultants showed significantly higher favorable ratings for learning IG in general hospital grand rounds than the trainees using the Chi-square test (*P* = 0.049). **The trainees showed significantly higher favorable ratings for learning IG in self-study/ exam preparations than consultants using the Chi-square test (*P* = 0.032).

**Figure 2.**
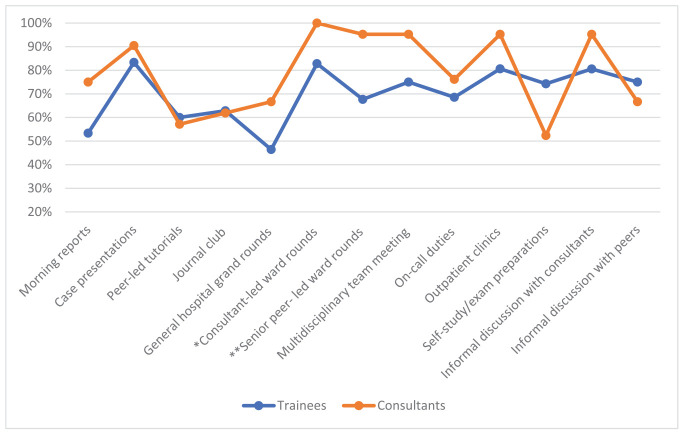
Management of complex clinical knowledge-favorable ratings. * The consultants showed significantly higher favorable ratings for learning CCK in consultant-led ward rounds than the trainees using the Chi-square test (*P* = 0.024). ** The consultants showed significantly higher favorable ratings for learning CCK in senior peer-led ward rounds than the trainees using the Chi-square test (*P* = 0.038).

The consultants preferred clinical learning opportunities for IG and CCK, with clinical rounds receiving the most favorable ratings (Figures 1 and 2, respectively). The trainees’ most favored learning opportunity for learning IG was self-study/exam preparations, which differed significantly from the consultants’ opinions (*P* = 0.032). The two groups’ opinions on perceived IG learning in general hospital grand rounds were also significantly different, with higher favorable ratings from consultants (*P* = 0.049). General hospital grand rounds also received the least favorable rating for CCK by trainees. The consultants rated all clinical rounds more favorably for learning CCK, with a significant difference from the trainees (*P* = 0.024 for consultant-led rounds and *P* = 0.038 for senior peer-led rounds).

The trainees and consultants favored a mixture of clinical, nonclinical, and informal learning opportunities for learning GH. None of the differences were statistically significant. The morning reports were rated as the least favored by both groups for learning GH (9/29 trainees, 31.03% and 7/20 consultants, 35%).

### Advanced CR Skills Learning in the Identified Learning Opportunities

**Figure 3.**
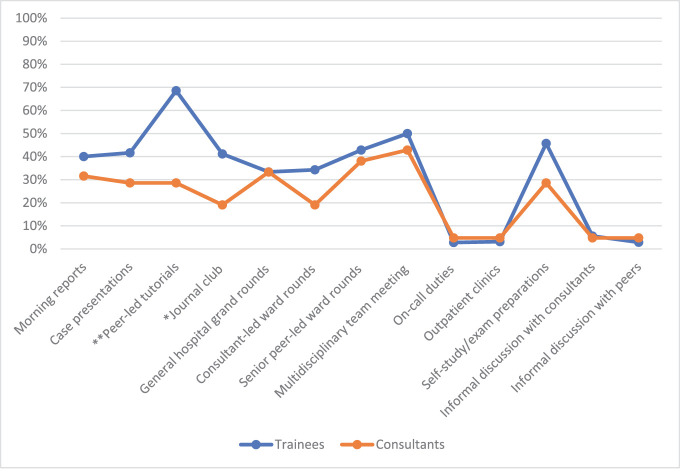
Quickly assign a diagnosis or management plan based on recognizing a pattern of clinical presentation-favorable ratings. * The trainees showed significantly higher favorable ratings for learning PR in the journal club than the consultants using the Chi-square test (*P* = 0.006). ** The trainees showed significantly higher favorable ratings for learning PR in peer-led tutorials than the consultants using the Chi-square test (*P* = 0.04).

**Figure 4.**
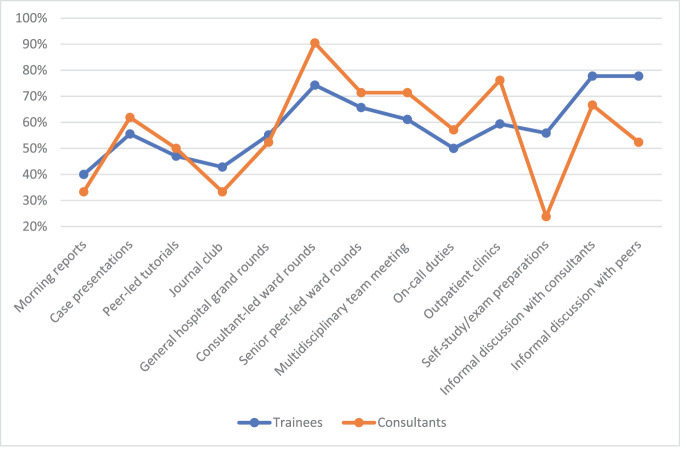
Cost-effective use of resources-favorable ratings.

**Figure 5.**
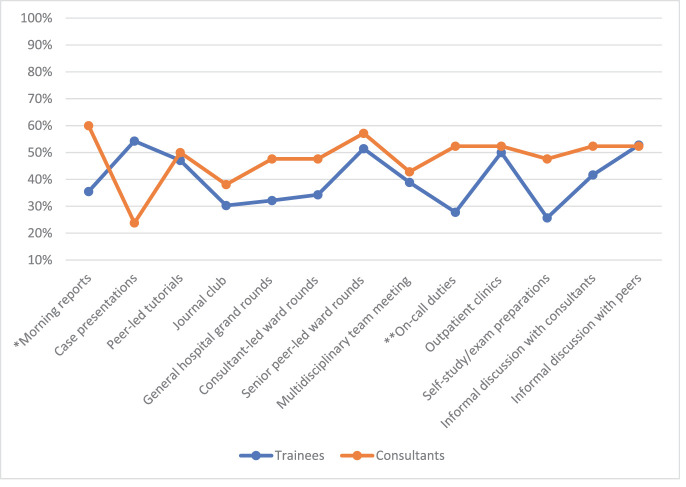
Recognition of the limits of evidence-based practice and the ability to communicate this to patients-favorable ratings. * The consultants showed significantly higher favorable ratings for learning EB in the morning reports than the trainees using the Chi-square test (*P* = 0.002). ** The consultants showed significantly higher favorable ratings for learning EB in on-call duties than the trainees using the Chi-square test (*P* = 0.031).

The groups’ perceived potential learning of advanced CR skills was less favorable than that of basic ones among the studied learning opportunities (Figures 3–5). The groups’ perceived learning of PR among all learning opportunities was lower than that of the other skills. PR was the only skill for which trainees rated learning opportunities more favorably than consultants. The trainees’ most favored learning opportunity for PR learning was peer-led tutorials, which the consultants rated significantly lower (*P* = 0.04). The trainees’ preferences for the journal club differed significantly from those of the consultants (*P* = 0.006).

The trainees’ most rated learning opportunity for CE was informal discussions with peers and consultants, whereas the consultants’ most rated learning opportunity was consultant-led ward rounds.

For EB learning, morning reports were the most favored by consultants, with a significant difference in trainees’ ratings (*P* = 0.002). The consultants’ ratings of on-call duties also differed significantly from those of the trainees (*P* = 0.031). The journal club received low ratings from both groups. Case presentations were most favored by trainees, while consultants ranked them the least favored.

## Discussion

The results provide insights into the two groups’ perspectives on CR learning. Both groups perceived the current learning opportunities as being less valuable for advanced skills that require higher cognitive processing. A detailed discussion of the two sets of CR skills follows.

### Basic Skills

Trainees preferred classroom and informal learning opportunities for IG and CCK, whereas consultants preferred clinical opportunities. The trainees’ preferences may reflect their limited awareness of the complexity of IG learning. They likely reduced IG learning to the content component and neglected the process. The preference for solitary learning activities may reflect a desire to control learning, which is challenging in the clinical environment. Trainees are probably more vulnerable to distressing cognitive overload in busy and high-demand clinical environments than in solitary or well-structured classrooms.^44^ Although IG, CCK, and GH are intertwined skills,^19^ preferences for learning GH differ from those for the other two skills. The consultants shared similar preferences with trainees regarding classroom learning opportunities. The agreement between the two groups may indicate their shared views on the intense contextual impact of cognitive overload on GH learning. Another factor that could explain the lower rating of GH compared with IG and CCK is that consultants and trainees may believe that learning about IG and CCK should be prioritized over GH at the trainees’ experience level. This may lead to unconscious efforts to promote teaching and learning these skills by modifying teaching styles.

### Advanced CR Skills

Clinical environments were not popular for the two groups in terms of learning advanced CR skills. The trainees again favored informal learning opportunities to learn CE, whereas the consultants favored classroom learning opportunities. The pattern of underestimating the potential benefits of the clinical environment and the preference for a *controlled learning environment* was also repeated with the EB skill. The lack of specific *management modules* likely contributes to informal, opportunistic, and non-standardized learning. Clinical environments can provide learning opportunities through modelling without formal modules. However, as discussed earlier, clinical environments are not the most favorable for learning CE. Classroom activities may provide a protected time that limits cognitive overload, but ward rounds are an excellent opportunity to create scientific curiosity and teach evidence translation to practice.^45–47^ The other concern about learning EB is the relatively low favorability of journal clubs despite being a classroom learning opportunity. This is a concern because the journal club is the only formal learning opportunity to teach evidence appraisal in local programs.

Although PR is the consultants’ primary CR method, the trainees favored self-study and peer-supported learning opportunities over consultant-led activities. The consultants themselves favored a mixture of learning opportunities that they did not dominate. The consultant-led ward rounds usually provide trainees with the most contact with consultants; hence, they are expected to serve as a good opportunity to learn the experts’ CR methods. However, neither group favored consultant-led ward rounds. This could be due to a preference for explicit teaching in consultant rounds, which may foster slower-paced analytical teaching. In addition, consultants might be discouraged from teaching PR if they try to accommodate the needs of the least skilled staff during the rounds. Residents’ short clinical placements with different subspecialties may also minimize exposure to diverse cases, which limits the development of rich mental patterns and non-analytical reasoning. The clinical cases must be organized in series to familiarize learners with typical cases first for mental exemplars to develop.^48^ However, controlling the clinical environment to organize cases in such a manner is unrealistic. This problem can be overcome by introducing similarity-based teaching to support PR processing.^49^

### The Least Favored Activities

Some learning opportunities were repeatedly perceived as contributing less favorably to learning CR skills. General hospital grand rounds, for example, were perceived less favorably by both groups in terms of IG, CCK, and EB skills. The trainees’ opinions were probably shaped by the current separation of the local mental health services’ organizational structure and delivery from other health services. Considering mental and physical illnesses as separate entities interferes with comprehensive person-centered care and hinders excellence in health care. Therefore, joint training sessions with other medical professionals are recommended.^50^ Morning reports, on-call duties, and linked learning opportunities received low favorable ratings. On-call duties likely create stressful learning environments during training. Trainees’ perceived learning of GH during on-call duties was poor despite good ratings for IG and CCK. The disconnected learning among the three skills may be explained by the trainees’ attempts to reduce stress and cognitive overload by depending on the consultant on call to generate clinical hypotheses from the gathered data. In addition, trainees may only invest in parts of the CR that meet the patient’s immediate needs rather than having a comprehensive CR. The on-call trainee presents all the cases in the morning report the following day. Owing to time pressure in morning reports, consultants’ CR processing likely depends on the non-analytical type, which trainees may find challenging to comprehend. This is probably the case with CE because the morning report is a forum for monitoring service usage and resource management.

The results showed significant differences in the perceptions of learning various CR skills between faculty and trainees. This difference highlights the possibility of an ineffective instructional design in some learning environments. Careful assessment of the learning environment, instructional design, and learner factors that impede the learning process is essential. Special attention should be paid to learning within clinical environments, as these are ranked the least favored for most CR skills. Cognitive overload can be managed by improving instructional designs^51^ and coaching trainees to manage stressful, high-demand environments.^52^ Introducing medical simulation sessions will likely offer opportunities for deliberate practice in a controlled environment that allows for graded exposure to real-life and high-demand environments.^53–55^ Postgraduate psychiatry programs may also consider introducing designated instructional designs for advanced CR skills to improve student learning. Local programs will also benefit from studying the actual learning of these skills and the impact of different contextual factors on CR cognition.

The relatively small sample size may have limited the results. In addition to job titles, no other demographic data were collected owing to the author’s limited resources. This may limit the readers’ ability to judge the external validity of the results. There were also missing questionnaire responses that were not statistically corrected. Missing responses may have biased the results. In addition, extreme opinions on the subject may be overrepresented because of questionnaire responder bias. This research only studied *perceived* learning, which may not reflect *actual* learning. However, perceived opinions are still necessary to understand learners’ educational needs.

## Conclusion

Despite the diversity of learning opportunities, they were perceived to facilitate the learning of basic CR skills more than opportunities that required higher cognitive processing. The preference for informal and classroom activities dominated the responses of the two groups but was more evident among trainees. This likely indicates that trainees are less able to manage contextual factors and experienced cognitive overload in the clinical environment. The preference for clinical learning opportunities decreased with the increased complexity of CR skills. Considering the lack of evidence of a superior single educational approach for CR teaching, this study highlights the need to implement multiple educational interventions, including coaching trainees, graded supervised exposure to varieties of complex clinical environments, clinical simulation and specific instructional designs targeting specific CR skills, etc., to reduce the impact of contextual factors on learner’s CR learning and processing. Faculty development programs are essential for improving CR teaching skills in complex clinical environments. This research opens the door for further assessments of *actual learning* in these learning opportunities, which will likely provide a comprehensive understanding of CR learning in training programs.

## List of Abbreviations

ACGME, Accreditation Council for Graduate Medical Education; CR, Clinical reasoning; CE, Cost-effective resource management skill; CVI, Content validity index; EB, Evidence-based practice skill; IG, Information gathering skill; ICV, Item content validity index; PR, Pattern recognition skill; LO, Learning opportunities; SVI, Scale validity index; UA, Universal expert agreement.

## Declarations

### Ethics Approval and Consent to Participate

The study received ethical approval from the HMC-IRB (protocol number: MRC-0-19-306) on 05/11/2019 and the University of Dundee School of Medicine Research Ethics Committee (SMED REC Number 19/ 176) on 9/12/2019 for the MSc dissertation.

### Availability of Data and Materials

The datasets used and/or analyzed during the current study are available from the corresponding author upon reasonable request.

### Conflict of Interests

The author declares that they have no conflict of interests.

## Acknowledgment

The author would like to thank Dr. Bonnie Lynch of the University of Dundee for her excellent supervision of the MSc thesis, of which this paper is part.

## Figures and Tables

**Table 1.** Basic and advanced clinical reasoning skills.

Basic CR skills	(1) To gather information (IG) (history taking, notes review, collateral information, nursing observation, multidisciplinary team (MDT) input, etc...)
	(2) To manage complex clinical knowledge (CCK) (e.g., organization, prioritization, and summarization of complex cases)
	(3) To generate and test clinical hypotheses taking into consideration the biological, psychological, and social factors that could influence the hypothesis (GH)
Advanced CR skills	(4) To quickly assign a diagnosis or management plan based on recognizing a pattern of clinical presentation (PR)
	(5) To manage resources cost-effectively (CE) (ordering tests, assigning observation based on the estimated risk level, consulting other medical specialties, etc.)
	(6) To recognize the limits of evidence-based practice and the ability to communicate this to patients (EB)

**Table 2.** Description of the learning opportunities.

**Learning opportunities**	**Description**
Classroom learning opportunities	
Morning reports	A daily classroom activity led by the on-call team and attended by the trainees and consultants. The on-call team presents the cases encountered during the on-call and discusses the management plan with the audience. The activity is primarily a platform to evaluate and manage hospital resource usage after hours. It is also considered a clinical and managerial learning educational platform.
Case presentations	A weekly classroom activity. The trainees take turns presenting and discussing one complex clinical case with the audience which is composed of other trainees and consultants. This activity is led by the clinical case’s treating consultant psychiatrist.
Peer-led tutorials	A weekly one-hour classroom activity. For each training year group, the trainees take turns moderating tutorials. The moderator decides on the methods of session delivery.
	The curriculum committee predetermines the tutorial topics. One of the consultant psychiatrists coaches the tutorial moderator.
Journal club	A weekly classroom learning opportunity led by a group of consultant psychiatrists. Trainees take turns presenting a journal article to the audience of trainees and some of the consultants.
General hospital grand rounds	A weekly classroom activity delivered in a general hospital. This activity is optional for psychiatrists. Every medical discipline takes turns presenting a topic to an audience of mixed medical fields.
Clinical learning opportunities	
Consultant-led ward rounds	Consultant-led ward rounds are strictly inpatient psychiatry units. Each team has consultant rounds around three times/week. The rounds are attended by the team’s consultant, senior and junior trainees, ward charge nurse, and medical and nursing students on occasion. The management plan of each patient is reviewed and updated during the round.
Senior peer-led ward rounds	The inpatient psychiatry unit’s senior trainee (clinical fellow) leads the senior peer-led ward rounds. The rounds are attended by the ward charge nurse, junior trainees, and, on occasion, medical and nursing students. The rounds occur around twice a week on the days when consultant rounds are not scheduled.
Multidisciplinary team meetings	A weekly clinical activity where each team, whether an inpatient or a community team, meets to discuss aspects of the clinical management of patients. Patients’ current needs are identified, and the management plan is updated. The team comprises disciplines like consultant psychiatrists, trainees, nurses, occupational therapists, psychologists, dietitians, physiotherapists, and social workers.
On-call duties	Clinical teams are expected to provide out-of-hours services for psychiatric emergencies. An on-call team consists of a junior resident who is based in the inpatient psychiatry units in the Mental Health Hospital, a senior resident based in the main general hospital and is expected to conduct consultation assessments in the emergency department, medical and surgical wards, a clinical fellow as a second on call, and a consultant psychiatrist as a third on call. The clinical fellow and the consultant are on call from home and may come to the hospital if required. The on-call system follows a hierarchy system in managing cases. According to the level of clinical complexity, the trainee may contact the doctor on the next on-call level.
Outpatient clinics	Junior residents work under the direct supervision of consultants in outpatient clinics. Senior residents and clinical fellows have clinics under their names and work under the indirect supervision of consultants. Senior trainees may discuss challenging outpatient cases with the consultants if required.
Informal learning opportunities	
Informal discussions with consultants and peers	Any clinical discussion occurs in social contexts and outside the learning opportunities mentioned above.
Self-study/exam preparations	Any self-directed learning is done by the trainees in response to intrinsic or extrinsic learning expectations.

## Footnotes

1 Clinical reasoning.

2 Information gathering skill.

3 Pattern recognition skill.

4 Cost-effective resource management skill.

5 Evidence-based practice skill.

6 Accreditation Council for Graduate Medical Education-International.

7 Accreditation Council for Graduate Medical Education.

8 Learning opportunities.

## Appendix 1

### Questionnaire 1

Below, we describe several clinical reasoning skills, along with the various learning opportunities that you might have to develop each skill. Considering your own experience, please rate the likelihood that each learning opportunity enhances the skills described.

For this research project, please consider Eva’s definition (2007, p.98) of clinical reasoning as **‘the ability to sort through a cluster of features presented by a patient and accurately assign a diagnostic label, with the development of an appropriate treatment strategy as the end goal’**.

**Table:** 1a: **Information gathering** (history taking, notes review, collateral information, nursing observation, multidisciplinary team input, etc.).

	**Very likely**	**Somewhat likely**	**Neutral**	**Somewhat unlikely**	**Very unlikely**	**Not applicable**
Morning reports						
Consultant-led ward rounds						
Near-peer (senior peer)-led ward rounds						
Multidisciplinary meeting						
Case presentations						
Journal club						
Peer-led tutorials						
On-call duties						
General hospital grand rounds						
Informal discussions with consultants						
Informal discussions with peers						
Outpatient clinics						
Self-study/Exam preparations						
Other (rate and describe in the box below)						

**Table:**

Other (please specify)						

**Table:** 1b: **Management of complex clinical knowledge** (organization, prioritization, and summarization of complex cases).

	**Very likely**	**Somewhat likely**	**Neutral**	**Somewhat unlikely**	**Very unlikely**	**Not applicable**
Morning reports						
Consultant-led ward rounds						
Near-peer (senior peer)-led ward rounds						
Multidisciplinary meeting						
Case presentations						
Journal club						
Peer-led tutorials						
On-call duties						
General hospital grand rounds						
Informal discussions with consultants						
Informal discussions with peers						
Outpatient clinics						
Self-study/Exam preparations						
Other (rate and describe in the box below)						

**Table:**

Other (please specify)						

**Table:** 1c: **Generation and testing of a clinical hypothesis, taking into consideration the biological, psychological, and social factors that could influence your hypothesis.**

	**Very likely**	**Somewhat likely**	**Neutral**	**Somewhat unlikely**	**Very unlikely**	**Not applicable**
Morning reports						
Consultant-led ward rounds						
Near-peer (senior peer)-led ward rounds						
Multidisciplinary meeting						
Case presentations						
Journal club						
Peer-led tutorials						
On-call duties						
General hospital grand rounds						
Informal discussions with consultants						
Informal discussions with peers						
Outpatient clinics						
Self-study/Exam preparation						
Other (rate and describe in the box below)						

**Table:**

Other (please specify)						

**Table:** 1d: **Cost-effective use of resources** (ordering tests, assigning observations based on the estimated level of risk, consulting other medical specialties, etc.).

	**Very likely**	**Somewhat likely**	**Neutral**	**Somewhat unlikely**	**Very unlikely**	**Not applicable**
Morning reports						
Consultant-led ward rounds						
Near-peer (senior peer)-led ward rounds						
Multidisciplinary meeting						
Case presentations						
Journal club						
Peer-led tutorials						
On-call duties						
General hospital grand rounds						
Informal discussions with consultants						
Informal discussions with peers						
Outpatient clinics						
Self-study/Exam preparations						
Other (rate and describe in the box below)						

**Table:**

Other (please specify)						

**Table:** 1e: **Quickly assigning a diagnosis or management plan based on recognizing a pattern of clinical presentation.**

	**Very likely**	**Somewhat likely**	**Neutral**	**Somewhat unlikely**	**Very unlikely**	**Not applicable**
Morning reports						
Consultant-led ward rounds						
Near-peer (senior peer)-led ward rounds						
Multidisciplinary meeting						
Case presentations						
Journal club						
Peer-led tutorials						
On-call duties						
General hospital grand rounds						
Informal discussions with consultants						
Informal discussions with peers						
Outpatient clinics						
Self-study/Exam preparations						
Other (rate and describe in the box below)						

**Table:**

Other (please specify)						

**Table:** 1f: **Recognition of the limits of evidence-based practice and the ability to communicate this to patients.**

	**Very likely**	**Somewhat likely**	**Neutral**	**Somewhat unlikely**	**Very unlikely**	**Not applicable**
Morning reports						
Consultant-led ward rounds						
Near-peer (senior peer)-led ward rounds						
Multidisciplinary meeting						
Case presentations						
Journal club						
Peer-led tutorials						
On-call duties						
General hospital grand rounds						
Informal discussions with consultants						
Informal discussions with peers						
Outpatient clinics						
Self-study/Exam preparations						
Other (rate and describe in the box below)						

**Table:**

Other (please specify)						

**Table:** Q2: Please add below any other clinical learning opportunities that improved your clinical reasoning skills.

Free text box

**Table:** Q3: Please indicate your level of training

First-year resident
Second-year resident
Third-year resident
Fourth- to fifth-year resident
First-year clinical fellow
Second-year clinical fellow
Third-year clinical fellow

## Appendix 2

### Questionnaire 2

We describe several clinical reasoning skills and various learning opportunities that may assist psychiatry doctors in developing each skill. Considering your experience as a clinical teacher, please rate the likelihood that each learning opportunity enhances the skills described.

For this research project, please consider Eva’s definition (2007, p.98) of clinical reasoning as **‘the ability to sort through a cluster of features presented by a patient and accurately assign a diagnostic label, with the development of an appropriate treatment strategy as the end goal’**.

**Table:** 1a: **Information gathering** (history taking, note review, collateral information, nursing observation, multidisciplinary team input, etc.).

	**Very likely**	**Somewhat likely**	**Neutral**	**Somewhat Unlikely**	**Very unlikely**
Morning reports					
Consultant-led ward rounds					
Near-peer (senior peer)- led ward rounds					
Multidisciplinary meeting					
Case presentations					
Journal club					
Peer-led tutorials					
On-call duties					
General hospital grand rounds					
Informal discussion with consultants					
Informal discussion with peers					
Outpatient clinics					
Self-study/Exam preparations					
Other (rate and describe in the box below)					

**Table:**

Other (please specify)					

**Table:** 1b. **Management of complex clinical knowledge** (organization, prioritization, and summarization of complex cases).

	**Very likely**	**Somewhat likely**	**Neutral**	**Somewhat unlikely**	**Very unlikely**
Morning reports					
Consultant-led ward rounds					
Near-peer (senior peer)-led ward rounds					
Multidisciplinary meeting					
Case presentations					
Journal club					
Peer-led tutorials					
On-call duties					
General hospital grand rounds					
Informal discussion with consultants					
Informal discussion with peers					
Outpatient clinics					
Self-study/Exam preparations					
Other (rate and describe in the box below)					

**Table:**

Other (please specify)					

**Table:** 1c. **To generate a clinical hypothesis and test it taking into consideration the biological, psychological, and social factors that could influence your hypothesis.**

	**Very likely**	**Somewhat likely**	**Neutral**	**Somewhat unlikely**	**Very unlikely**
Morning reports					
Consultant-led ward rounds					
Near-peer (senior peer)- led ward rounds					
Multidisciplinary meeting					
Case presentations					
Journal club					
Peer-led tutorials					
On-call duties					
General hospital grand rounds					
Informal discussion with consultants					
Informal discussion with peers					
Outpatient clinics					
Self-study/Exam preparations					
Other (rate and describe in the box below)					

**Table:**

Other (please specify)					

**Table:** 1d. **Cost-effective use of resources** (ordering tests, assigning observations based on the estimated level of risk, consulting other medical specialties, etc.).

	**Very likely**	**Somewhat likely**	**Neutral**	**Somewhat unlikely**	**Very unlikely**
Morning reports					
Consultant-led ward rounds					
Near-peer (senior peer)-led ward rounds					
Multidisciplinary meeting					
Case presentations					
Journal club					
Peer-led tutorials					
On-call duties					
General hospital grand rounds					
Informal discussion with consultants					
Informal discussion with peers					
Outpatient clinics					
Self-study/Exam preparations					
Other (rate and describe in the box below)					

**Table:**

Other (please specify)					

**Table:** 1e. **Quickly assign a diagnosis or management plan based on recognizing a pattern of clinical presentation.**

	**Very likely**	**Somewhat likely**	**Neutral**	**Somewhat unlikely**	**Very unlikely**
Morning reports					
Consultant-led ward rounds					
Near-peer (senior peer)- led ward rounds					
Multidisciplinary meeting					
Case presentations					
Journal club					
Peer-led tutorials					
On-call duties					
General hospital grand rounds					
Informal discussion with consultants					
Informal discussion with peers					
Outpatient clinics					
Self-study/Exam preparations					
Other (rate and describe in the box below)					

**Table:** 1f. **Recognizing the limits of evidence-based practice and the ability to communicate this to patients.**

	**Very likely**	**Somewhat unlikely**	**Neutral**	**Somewhat unlikely**	**Very unlikely**
Morning reports					
Consultant-led ward rounds					
Near-peer (senior peer)-led ward rounds					
Multidisciplinary meeting					
Case presentations					
Journal club					
Peer-led tutorial					
On-call duties					
General hospital grand rounds					
Informal discussion with consultants					
Informal discussion with peers					
Outpatient clinics					
Self-study/Exam preparations					
Other (rate and describe in the box below)					

**Table:**

Other (please specify)					

**Table:** Q2: Please add below any other clinical learning opportunities you consider essential for the trainees to learn clinical reasoning skills.

Free text box

**Table:** Q3: Please indicate your job title:

Senior consultant psychiatrist
Consultant psychiatrist
Associate consultant psychiatrist
Psychiatry specialist

**Table:** Q4: Please indicate your years of experience in psychiatry:

4-10 years
11-20 years
> 20 years

## References

[1] Gabriel A, Violato C (2013). Problem-solving strategies in psychiatry: differences between experts and novices in diagnostic accuracy and reasoning. Adv Med Educ Pract.

[2] Linsen A, Elshout G, Pols D, Zwaan L, Mamede S (2017). Education in clinical reasoning: an experimental study on strategies to foster novice medical students’ engagement in learning activities. Health Profess Educ.

[3] Loveday T, Wiggins M, Festa M, Schell D, Twigg D, Latorre Carmona P, Sánchez J, Fred A Pattern recognition as an indicator of diagnostic expertise. Pattern Recognition - Applications and Methods. Advances in Intelligent Systems and Computing.

[4] Norman G, Young M, Brooks L (2007). Non-analytical models of clinical reasoning: The role of experience. Med Educ.

[5] Norman G (2005;). Research in clinical reasoning: past history and current trends. Med Educ.

[6] Robertson LJ (1996). Clinical reasoning, part 2: novice/expert differences. Br J Occup Therapy.

[7] Jensen G, Resnik L, Haddad A, Higgs J, Jones M (2008). Expertise and clinical reasoning.

[8] Süß HM, Oberauer K, Wittmann WW, Wilhelm O, Schulze R (2002;). Working-memory capacity explains reasoning ability - And a little bit more. Intelligence.

[9] Hruska P, Krigolson O, Coderre S, McLaughlin K, Cortese F, Doig C (2016). Working memory, reasoning, and expertise in medicine—insights into their relationship using functional neuroimaging. Adv Health Sci Educ.

[10] Rotgans JI, Schmidt HG, Rosby LV, Tan GJS, Mamede S, Zwaan L (2019;). Evidence supporting dual-process theory of medical diagnosis: a functional near-infrared spectroscopy study. Med Educ.

[11] Konopasky A, Ramani D, Ohmer M, Battista A, Artino AR, McBee E (2020). It totally possibly could be: how a group of military physicians reflect on their clinical reasoning in the presence of contextual factors. Mil Med.

[12] Pelaccia T, Tardif J, Triby E, Charlin B (2011;). An analysis of clinical reasoning through a recent and comprehensive approach: the dual-process theory. Med Educ Online.

[13] Choi HH, van Merriënboer JJG, Paas F (2014;). Effects of the physical environment on cognitive load and learning: towards a new model of cognitive load. Educ Psychol Rev.

[14] Réa-Neto A (1998). Clinical reasoning--the diagnostic and therapeutic decision process. Rev Assoc Med Bras (1992).

[15] McBee E, Ratcliffe T, Picho K, Schuwirth L, Artino AR, Yepes-Rios AM (2017). Contextual factors and clinical reasoning: Differences in diagnostic and therapeutic reasoning in board certified versus resident physicians. BMC Med Educ.

[16] Harendza S, Krenz I, Klinge A, Wendt U, Janneck M (2017). Implementierung eines clinical-reasoning-kurses im PJ-tertial innere medizin und dessen wirkung auf studentische fähigkeiten der fallpräsentation und der differentialdiagnostik. GMS J Med Educ.

[17] Haring C, Cools B, Van Gurp P, Van Der Meer J, Postma C (2017). Observable phenomena that reveal medical students’ clinical reasoning ability during expert assessment of their history taking: A qualitative study. BMC Med Educ.

[18] Silverman J, Brown J, Noble LM, Papageorgiou A, Kidd J (2015). Information gathering and clinical reasoning. Clinical Communication in Medicine.

[19] Nardone DA (1990). Collecting and Analyzing Data: Doing and Thinking. Clinical Methods: The History, Physical, and Laboratory Examinations.

[20] Ark TK, Brooks LR, Eva KW (2006;). Giving learners the best of both worlds: Do clinical teachers need to guard against teaching pattern recognition to novices?. Acad Med.

[21] Frank J, Snell L, Sherbino J (2015). CanMEDS 2015 Physician Competency Framework. CanMEDS 2015 Physician Competency Framework.

[22] The General Medical Council (2012). Recognising and approving trainers: the implementation plan.

[23] Vize R (2015;). Why doctors don’t dare go into management. Bmj.

[24] Emparanza JI, Cabello JB, Burls AJE (2015). Does evidence-based practice improve patient outcomes? An analysis of a natural experiment in a Spanish hospital. J Eval Clin Pract.

[25] Connor L, Dean J, McNett M, Tydings DM, Shrout A, Gorsuch PF (2023). Evidence-based practice improves patient outcomes and healthcare system return on investment: Findings from a scoping review. Worldviews Evid Based Nurs.

[26] Shah D, Sachdev H (2007). Evidence-based medicine. Indian J Orthop.

[27] Rencic J (2011;). Twelve tips for teaching expertise in clinical reasoning. Med Teach.

[28] Schmidt HG, Mamede S (2015). How to improve the teaching of clinical reasoning: a narrative review and a proposal. Med Educ.

[29] Torre D, Durning SJ, Rencic J, Lang V, Holmboe E, Daniel M (2020). Widening the lens on teaching and assessing clinical reasoning: from “in the head” to “out in the world.”. Diagnosis.

[30] Guraya SY (2016;). The pedagogy of teaching and assessing clinical reasoning for enhancing the professional competence: a systematic review. Biosci Biotechnol Res Asia.

[31] Arias D, Saxena S, Verguet S (2022). Quantifying the global burden of mental disorders and their economic value. EClinicalMedicine.

[32] GBD 2019 Mental Disorders Collaborators (2022). Global, regional, and national burden of 12 mental disorders in 204 countries and territories, 1990-2019: a systematic analysis for the Global Burden of Disease Study 2019. Lancet Psychiatry.

[33] Bener A, Abou-Saleh M, Dafeeah E, Bhugra D (2015;). The prevalence and burden of psychiatric disorders in primary health care visits in Qatar: too little time?. J Family Med Prim Care.

[34] Ministry of Public Health (2014). Mental Healthcare Act 2014.

[35] Albahari D (2023). Learning Clinical Reasoning: The Experience of Postgraduate Psychiatry Trainee Doctors in Qatar. Teach Learn Med.

[36] Vidyarthi AR, Kamei R, Chan K, Goh SH, Lek N (2015). Factors associated with medical student clinical reasoning and evidence based medicine practice. Int J Med Educ.

[37] Cuthbert L, Teather D, Teather B, Sharples M, DuBoulay G (1999;). Expert / Novice differences in Diagnostic Medical Cognition - A Review of the Literature. Computing.

[38] Schmidt HG, Norman GR, Boshuizen HPA (1990;). A cognitive perspective on medical expertise: Theory and implications. Academic Medicine.

[39] Bandura A (1989). Human Agency in Social Cognitive Theory. Am Psychol.

[40] Torre D, Durning SJ (2015). Social cognitive theory: thinking and learning in social settings. Researching Medical Education [Internet].

[41] Brown JS, Collins A, Duguid P (1989;). Situated Cognition and the Culture of Learning. Educational Researcher.

[42] Wilson AL (1993). The promise of situated cognition. New Dir Adult Continuing Educ.

[43] SurveyMonkey (2018). SurveyMonkey: The world’s most popular free online survey tool. SurveyMonkey.

[44] de Jong T (2010). Cognitive load theory, educational research, and instructional design: some food for thought. Instr Sci.

[45] Houchens N, Harrod M, Fowler KE, Moody S, Saint S (2017). How Exemplary Inpatient Teaching Physicians Foster Clinical Reasoning. Am J Med.

[46] Hatala R, Keitz SA, Wilson MC, Guyatt G (2006). Beyond journal clubs: Moving toward an integrated evidence-based medicine curriculum. J Gen Intern Med [Internet].

[47] Macdonald H (2011). Are journal clubs an essential tool for postgraduate education?. Bmj.

[48] Pelaccia T, Tardif J, Triby E, Charlin B (2011;). An analysis of clinical reasoning through a recent and comprehensive approach: The dual-process theory. Med Educ Online.

[49] Jia Z, Zeng X, Duan H, Lu X, Li H (2020). A patient-similarity-based model for diagnostic prediction. Int J Med Inform.

[50] Byrne L, Goslar D, James A, Khan M, Lovett K, David Morris P (2018). Person-centred care: implications for training in psychiatry Person-Centred Training and Curriculum (PCTC) Scoping Group Special Committee on Professional Practice and Ethics Acknowledgements.

[51] Leppink J (2017). Cognitive load theory: Practical implications and an important challenge. J Taibah Univ Med Sci.

[52] Szulewski A, Howes D, van Merriënboer JJG, Sweller J (2021). From theory to practice: the application of cognitive load theory to the practice of medicine. Acad Med.

[53] Vyas D, Ottis EJ, Caligiuri FJ (2011). Teaching clinical reasoning and problem-solving skills using human patient simulation. Am J Pharm Educ.

[54] Mutter MK, Martindale JR, Shah N, Gusic ME, Wolf SJ (2020). Case-based teaching: does the addition of high-fidelity simulation make a difference in medical students’ clinical reasoning skills?. Med Sci Educ.

[55] Watari T, Tokuda Y, Owada M, Onigata K (2020;). The utility of virtual patient simulations for clinical reasoning education. Int J Environ Res Public Health.

